# The qualified presumption of safety assessment and its role in EFSA risk evaluations: 15 years past

**DOI:** 10.1093/femsle/fny260

**Published:** 2018-12-10

**Authors:** Lieve Herman, Marianne Chemaly, Pier Sandro Cocconcelli, Pablo Fernandez, Günter Klein, Luisa Peixe, Miguel Prieto, Amparo Querol, Juan Evaristo Suarez, Ingvar Sundh, Just Vlak, Sandra Correia

**Affiliations:** 1 European Food Safety Authority (EFSA) Panel on Biological Hazards (BIOHAZ); 2 BIOCONTAM BIOHAZ WG on the update of the list of QPS recommended biological agents intentionally added to food or feed as notified to EFSA (2017-19) (M-2016-0211); 3 French Agency for Food, Environmental and Occupational Health Safety (Anses), Laboratory of Ploufragan-Plouzané, Unit of Hygiene and Quality of Poultry and Pork Products (UHQPAP), BP 53, 22440 Ploufragan, France; 4 DiSTAS, Università Cattolica del Sacro Cuore, Piacenza, Via Emilia Parmense 84, 29121 Piacenza, Italy; 5 Polytechnic University of Cartagena, Department of Food Engineering and E.A., Paseo Alfonso XIII 48, 30203 Cartagena, Spain; 6 UCIBIO/REQUIMTE, Laboratório de Microbiologia, Faculdade de Farmácia, Universidade do Poro, Rua Jorge Viterbo Ferreira n° 228. 4050-313 Porto. Portugal; 7 Institute of Food Science and Technology, Campus de Vegazana, s/n, University of León, 24007 León, Spain; 8 Food Biotechnology Department, Systems Biology in Yeast of Biotechnological Interest, Instituto de Agroquímica y Tecnología de los Alimentos, IATA-CSIC, C/ Catedrático Agustín Escardino Benlloch, 7, E-46980 Paterna - Valencia - Spain; 9 Área de Microbiología, Facultad de Medicina, Universidad de Oviedo, Julián Clavería 6, 33006 Oviedo, Spain; 10 Swedish University of Agricultural Sciences (SLU), Department of Molecular Sciences, PO Box 7015, SE-75007 Uppsala, Sweden; 11 Laboratory of Virology, Wageningen University & Research, Droevendaalsesteeg 1, 6708 PD Wageningen, The Netherlands; 12 Unit of Biological Hazards and Contaminants (BIOCONTAM), European Food Safety Authority (EFSA), via Carlo Magno 1A, 43126 Parma, Italy

**Keywords:** qualified presumption of safety, EFSA safety assessment, QPS list, QPS opinion, QPS statement

## Abstract

Microorganisms are intentionally added at different stages of the food and feed chain (food or feed additive, novel food or plant protection product) and are subjected to regulation and safety assessment by the European Food Safety Authority. Safety evaluation is based on application dossiers for market authorisation to the European Commission. The qualified presumption of safety (QPS) concept was developed in 2003^1^ to provide a harmonised generic safety pre-appraisal of the above microorganisms. Unambiguously defined biological taxonomic units (TUs) are assessed for their body of knowledge, their safety and their end use. Identified safety concerns for a certain TU can be, where reasonable in number and not universally present, reflected as ‘qualifications.’ Strains belonging to TUs having QPS status may benefit of a fast track evaluation. The lowest TU for which the QPS status is granted is the species level for bacteria and yeasts and the family for viruses. The QPS concept is also applicable to genetically modified microorganisms used for production purposes. Based on the current body of knowledge and/or the ambiguous taxonomic position, some TUs, such as filamentous fungi, bacteriophages, *Enterococcus faecium*, *Escherichia coli*, *Streptomyces* spp. and *Oomycetes*, are not considered liable for QPS status.

## INTRODUCTION

The European Food Safety Authority (EFSA) is a European agency funded by the European Union. EFSA operates independently of the European legislative and executive institutions (Commission, Council, Parliament) and EU Member States to provide scientific advice and perform risk assessment and communication of issues related to the safety of food and feed and their possible impact on the environment (biodiversity of plant and animal habitats). A wide variety of microorganisms and viruses are intentionally added at different stages of the food and feed chain and are subjected to regulation and safety assessment by EFSA. They can be a food or feed additive, a novel food or a plant protection product. Safety evaluation is based on an application dossier for market authorisation to the European Commission. The qualified presumption of safety (QPS) assessment was developed to provide a harmonised generic safety pre-assessment to support the risk assessments performed by EFSA’s scientific Panels and Units (Leuschner *et al.*^2010^).

As stated in 2007 by EFSA (EFSA 2007), a safety assessment of a biological taxonomic unit (TU) can be made based on four pillars [taxonomic identification, body of knowledge, possible pathogenicity (‘safety concerns’) and end use]. If the TU did not raise safety concerns or if any safety concerns could be defined and excluded (the qualification), the TU could be granted QPS status. Thereafter, any strain of microorganism whose identity could be unambiguously established and assigned to a TU with QPS status would be freed from the need for further assessment other than satisfying any qualification specified. Microorganisms not considered suitable for a QPS status would remain subject to a full safety assessment.

TUs recommended for the QPS status are incorporated into the QPS list. The list, first established in 2007 (EFSA 2007), has been periodically revised and updated. Based on repeated reviews of the scientific literature, a TU could be excluded from the list if new safety concerns would be identified. Since 2014, every 3 years, the updated QPS list is published through a Scientific Opinion, adopted by the Panel on Biological Hazards (BIOHAZ) after drafting by the working group on QPS (QPS WG). Intermediate deliverables in the form of Panel Statements are produced and published covering periods of around 6 months. These statements include both the evaluation of new TUs for a possible QPS status and the results of the monitoring through extended literature searches of possible safety concerns related to those TUs already on the QPS list. The Opinion and the Panel Statements, including the QPS^3^ list, are published online in the EFSA journal.

### QPS: definition and assessment

QPS provides a safety status for microorganisms intentionally used in the food and feed chain. These microorganisms can be used as living entities that may reach the consumer as such, or may be used as production organisms or as dead biomass and, in this case, no viable cells should be found in the final product. The lowest TU for which the QPS status is granted is the species level for bacteria and yeasts and the family for viruses. In the case of genetically modified microorganisms (GMMs), for which the species of the recipient strain qualifies for the QPS status, and for which the genetically modified state does not give rise to safety concerns, the QPS approach can be extended to genetically modified production strains (EFSA BIOHAZ Panel *et al.*2018a). The possible effect of the genetic modification on the safety of the product is assessed for each genetically modified strain by the respective EFSA unit. Guidance for safety evaluation at the strain level is described by EFSA FEEDAP Panel *et al.* (2018).

For each TU the following aspects are assessed:

#### Taxonomic identification

Only unambiguously defined biological TUs are considered for inclusion in the QPS list. Taxonomic identity is based on the internationally accepted classification of the List of Prokaryotic Names with Standing in Nomenclature (LPSN; Euzeby 2018) and the modifications that appear in the *International Journal of Systematic and Evolutionary Microbiology* (IJSEM; Oren and Garrity, 2015). The nomenclature and taxonomy of fungi, including yeasts, are covered by the International Code of Nomenclature for Algae, Fungi, and Plants (Turland *et al*. 2018). The taxonomy and nomenclature of viruses are the responsibility of the International Committee on Taxonomy of Viruses (ICTV 2018), which publishes regular updates online. As such, accurate taxonomy is one of the cornerstones of the QPS concept. Microbiological taxonomy is under constant review, a process expected in the near future to be gradually further completed and changed based on whole genome sequence data. This re-classification of microorganisms will lead to necessary adaptations in the QPS list of microorganisms, updated in each QPS statement.

#### Body of knowledge

The body of knowledge includes the history of use (Constable *et al.*^2007^; Pariza *et al.*^2015^), the ecology in the natural environment, clinical aspects, industrial applications, special properties, etc. Properties related to colonisation ability and routes for dispersal are considered. Knowledge about the interactions with other microorganisms, especially with respect to antagonism and competitive ability, is also relevant. The body of knowledge is investigated based on the scientific literature. This includes peer-reviewed papers published in journals and books that appear in scientific literature databases. The articles identified through the extensive literature searches on pre-selected databases with standardized key words are screened and evaluated by QPS WG members.

It is deemed necessary that there is enough scientific evidence to evaluate all the relevant aspects to be considered for the intended end use. If this information is not available, the QPS status is not granted, due to ‘lack of sufficient body of knowledge.’

#### Safety concerns

Safety concerns relate to the possible presence of virulence factors that may contribute to the pathogenicity of the microorganism to humans and animals, and to the possible production of biologically active substances, such as antimicrobials and toxins. Relevant information includes case reports of human disease caused by the TU. The assessment takes into account whether the negative impacts affected patients with severe underlying diseases or immunosuppression, and whether transmission occurred through food or other routes (e.g. medical devices). Reports of diseases on livestock and wild animals and whether diseases occur through feed or other routes (e.g. wounds, inhalation) are also relevant for identifying potential safety concerns.

Because each bacterial TU can harbour strains with acquired antimicrobial resistance genes conferring resistance to clinically relevant antimicrobials, the absence of any of these genes is considered as a general qualification for all bacterial TUs on the QPS recommended list and has to be assessed at the strain level (see further). Intrinsic antimicrobial resistance is not considered as a risk.

The assessment of environmental safety considers information on the capability of the TU to survive, compete and proliferate in the environment. The possibility that it may cause adverse health or environmental effects to animals and plants is considered when it is not directly connected to pathogenicity and infectivity to vertebrate animals. Contained food and feed production facilities are considered to have a safe set-up.

#### End use

The body of knowledge and the safety concerns may differ for the living organisms and for the dead biomass or specific compounds produced. Usually, the QPS approach assesses the deliberate introduction of viable microorganisms with consequent exposure of humans and/or animals. The second circumstance involves only the products derived from microbial metabolism, such as cell-free extracts in the case of enzymes, vitamins and amino acids. In this latter case, the QPS recommendation may only apply to this specific end use not including living organisms, which is indicated as a qualification in the QPS list (see further).

Some aspects are not covered by the QPS concept:

- hazards linked to the formulation or processing of the products;
- hazards linked to allergenicity to residual microbial components; nevertheless, if there is science-based evidence related to well-defined clinical cases, this is taken into consideration;
- potential environmental impact of microorganisms and viruses used for plant protection purposes;
- potential harm to users and workers derived from handling of the product (e.g. dermal, inhalation, ingestion).

Based on an insufficient body of knowledge, an ambiguous taxonomic position and/or a general ability of the TU to produce biologically active compounds that might be deleterious for humans, animals or the environment, some TUs are not granted with QPS status (EFSA BIOHAZ Panel *et al.*^2017^):

- filamentous fungi due to wide distribution of gene clusters encoding secondary metabolites that are usually strain linked and whose products possess diverse biological activities, including toxigenicity;
- bacteriophages—due to the fact that the lowest level taxonomic level is the order, which is considered too wide and that the assessment of transducing potential has to be performed at the individual phage type level;
- *Enterococcus faecium*—because with the available information the safety status attribution at the species level is not possible and the pathogenic potential of the strains in relation to combinations of putative virulence markers is still not clarified. Guidance for safety evaluation at the strain level is described by EFSA FEEDAP Panel (2018);
- *Escherichia coli*—due to the variable content on virulence features within members of this species;
- *Streptomyces* spp.—due to the ability for production of secondary metabolites not all yet identified and varying on a strain basis;
- *Oomycetes—*due to insufficient knowledge about toxigenic potential and the unknown activity of many of their secondary metabolites.

### QPS: qualifications

Identified safety concerns for a TU can be, where reasonable in number and not universally present, reflected as ‘qualifications.’ For a specific strain belonging to a TU with QPS status, any qualification needs to be evaluated by the relevant EFSA Unit based on the information provided in the respective dossier.

Absence of ‘acquired antimicrobial resistance genes to clinically relevant antimicrobials’ is a generic qualification for bacterial TUs. The verification that a specific bacterial strain fulfils this qualification is conducted by the specific EFSA Unit, to which the notification was assigned. Within the framework of EFSA activities, the use of interpretative criteria and methods to define and monitor antimicrobial resistance have been harmonized (EFSA BIOHAZ Panel *et al.*^2017^).

‘Absence of toxigenic activity’ for *Bacillus* spp. is based on the notion that some strains among the *Bacillus* species on the QPS list have caused foodborne intoxication in the past, which has been attributed to the production of compounds with toxic activity. Technical guidance to identify these toxic compounds among *Bacillus* species has been elaborated (EFSA FEEDAP Panel 2014).

The qualification ‘for production purpose only’ applies to TUs used for the biosynthesis of specific products for use in the food chain and subject to specific authorisation (e.g. food processing enzymes and feed and food additives such as vitamins, amino acids, polysaccharides and enzymes). For most of the TUs used for production, data are lacking on direct exposure to humans and animals, while there is a long history of use of their fermentation products in the food chain. This qualification implies the absence of viable cells of the production organism in the final product and is also applicable to food and feed products based on ‘dead’ biomass of the micro-organism (EFSA BIOHAZ Panel *et al.*2018b).

### QPS and EFSA risk assessment

**Figure 1.**
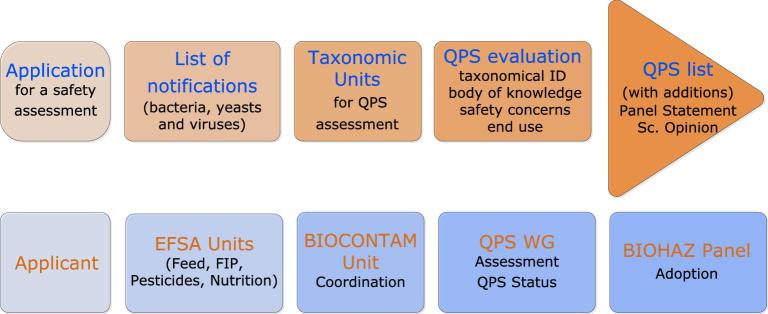
Workflow diagram describing how the QPS assessment is triggered by an application for market authorisation of a regulated product.

The safety assessment is based on an application dossier introduced to the individual EFSA Panels/Units by the respective services of the European Commission or Competent Authority in member states (Fig. 1). The EFSA Panels/Units currently involved in the assessment of regulated products that may involve the use of microorganisms, are:

#### Feed additives panel (FEEDAP)

Responsible for risk assessment of microorganisms used as feed additives by means of viable organisms or as production organism for feed additives, as defined in Regulation (EC) 1831/2003.

#### Food contact materials, enzymes and processing aids panel (CEP)

Responsible for the risk assessment of food enzymes, food amino acids and food ingredients in agreement with the QPS approach that entered EU law with the publication of a new Commission Implementing Regulation (EU) No 562/2012 amending Commission Regulation (EU) No 234/2011 for food applications.

#### Nutrition, novel foods and food allergens panel (NDA)

Responsible for the safety assessment of novel foods that fall under Regulation (EU) No 2283/2015. In the framework of Regulation (EC) No 1924/2006 on health claims made on foods (including microorganisms), the NDA Panel is also responsible for verifying the scientific substantiation (efficacy assessment) of submitted health claims. Under this framework, it should be noted that a safety assessment is not foreseen. However, where relevant, the NDA Panel may recommend also, in the case of health claims, restrictions of use based on safety considerations.

#### Pesticides unit

Responsible for the peer review of microbial plant protection products that are submitted for approval under Regulation (EC) No 1107/2009. For microorganisms intended as active agents of plant protection products, the ‘rapporteur member state’ has the main responsibility for performing a risk assessment and EFSA (the Pesticides Unit) performs a peer review of the risk assessment of the active agent/organism.

Although many species of food starter cultures are on the QPS list, it has to be noted that use of microorganisms in food fermentations is not regulated at the EU level and as such food starter cultures are not subjected to a safety assessment by EFSA. After the establishment of the first QPS list, no new starter organisms have been included because they are not subject of a notification to EFSA for market authorization (see for further explanation the division ‘QPS: history and workflow within EFSA’).

**Figure 2.**
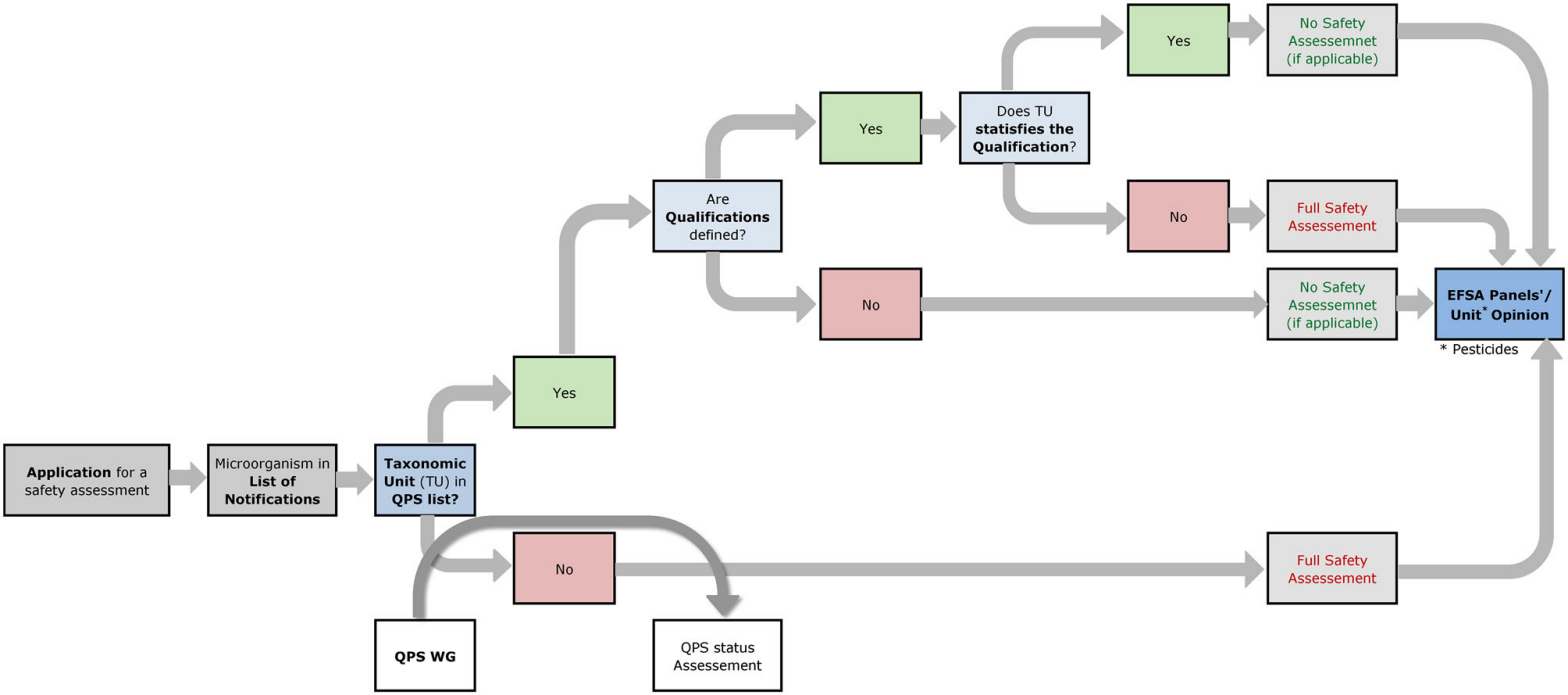
Workflow diagram describing how EFSA Units incorporate the qualified presumption of safety (QPS) status of a certain taxonomic unit (TU) into the safety assessment process of a microorganism notified through an application for market authorisation (adapted from EFSA BIOHAZ Panel et al. 2017). Possible qualifications of QPS microorganisms need to be evaluated by the relevant EFSA Unit based on the information provided in the respective dossier. The specific safety assessment is included in the EFSA Unit's Opinion and reference to the QPS status of the TU notified and eventual qualifications are included in that Opinion.

Strains of TUs with QPS status still require an assessment based on the individual data package sent to the respective EFSA unit. However, a fast track evaluation can be employed, with less requirements in relation to the risks that might be associated with the microorganism (see Fig. 2). Two examples are: (i) if the strain is to be used for production of a food enzyme, the application does not need to include specific toxicological test data (Commission Implementing Regulation (EU) No 562/2012); (ii) if the strain is intended for the production of a feed additive or as a living organism for animal performance improvement, no assessment of the safety for the animal target species, consumer or the environment is required. The data required for organisms with QPS status are in both cases limited to the confirmation of the unambiguous identification of the strain and to the confirmation that any qualifications are met. Guidance for assessing these requirements is provided by EFSA (EFSA FEEDAP Panel *et al.*^2018^). This guidance is adopted by the FEEDAP Panel but is also used for the evaluation of microorganisms in the frame of an application of other areas covered by other EFSA Units.

### QPS: history and workflow within EFSA

**Figure 3.**
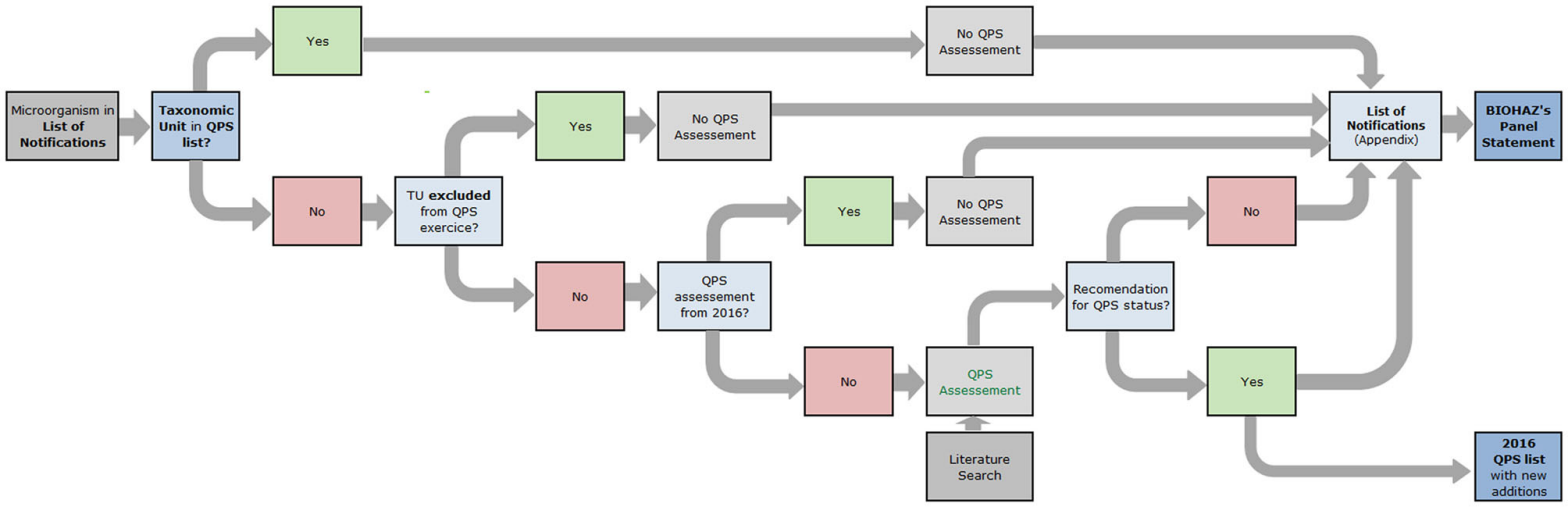
Workflow diagram describing how the evaluation of newly notified taxonomic units (TUs), depending if it is or not found in the qualified presumption of safety (QPS) list, is included in each BIOHAZ Panel Statement (adapted from EFSA BIOHAZ Panel et al. 2017). EFSA Units update the ‘List of Notifications’ (Fig. 1), and for each period of 6 months, EFSA includes them in an appendix of the on-going Panel Statement and checks the respective TU and chooses which are to be considered for the QPS status assessment. If a new TU receives a QPS recommendation (and possible qualifications), it is included in the valid QPS list.

**Figure 4.**
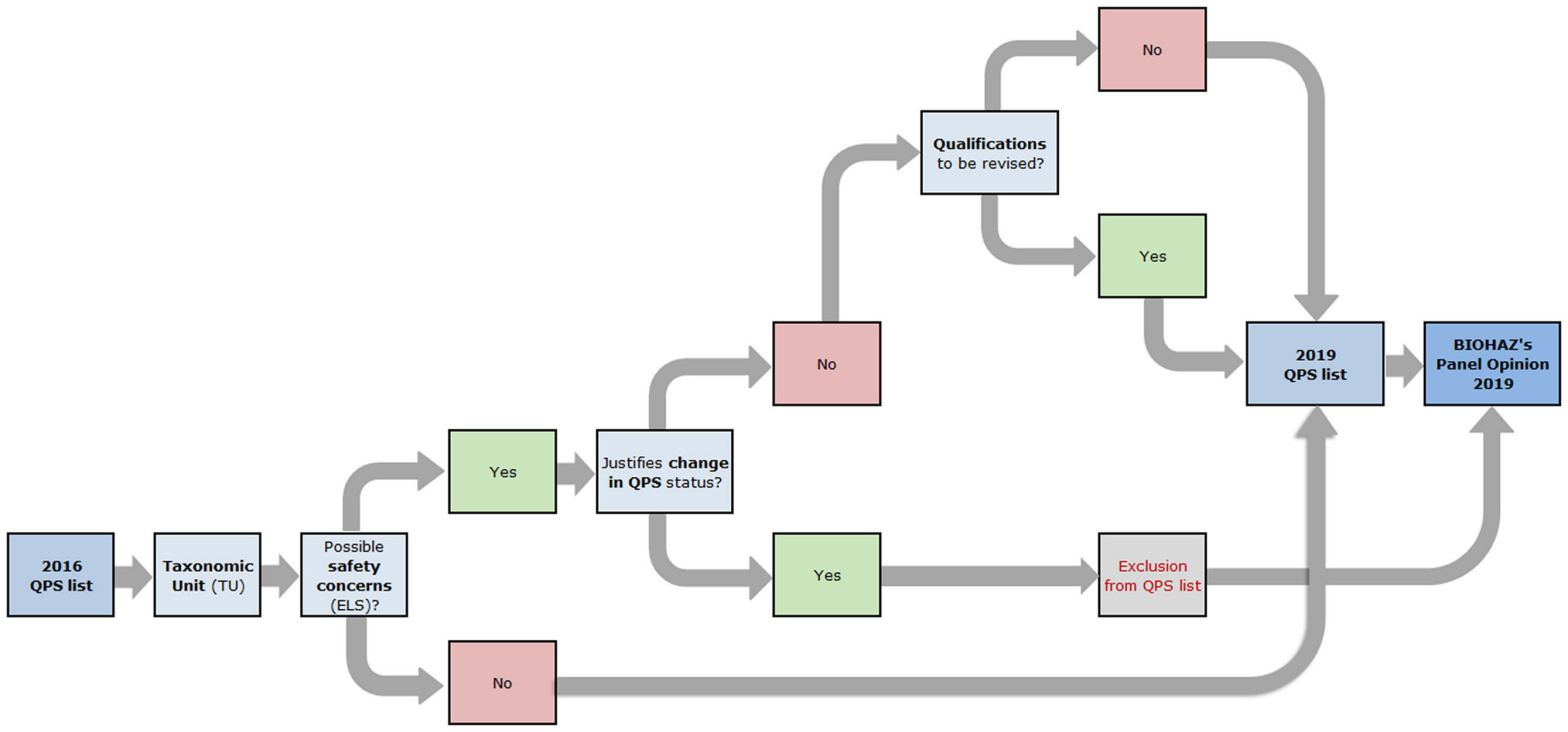
Workflow diagram describing how the BIOHAZ qualified presumption of safety (QPS) list is maintained and the QPS Opinion is prepared (adapted from EFSA BIOHAZ Panel et al. 2017). The QPS Opinion contains an update of the QPS list and the results of the 3-years Extended Literature Search (ELS) on the QPS taxonomic units (TUs), together with an update of the QPS process.

The first QPS list was prepared by the EFSA Scientific Committee and published in 2007. It was the result of the safety assessment of microorganisms likely to be the subject of an EFSA Opinion, the majority being the result of notifications to EFSA for market authorisation as sources of food and feed additives, food enzymes and plant protection products, but, at that moment, introduced independently of any particular application (EFSA 2007). From 2008 onwards, the further updates of the QPS list have been performed by the EFSA BIOHAZ Panel, only assessing TUs in the frame of new notifications of microorganisms through application of the corresponding dossiers arriving in EFSA. From 2014, the process includes the publication of a QPS Panel Statement every 6 months (Fig. 3) and a QPS Opinion every 3 years (Fig. 4) EFSA BIOHAZ Panel et al. 2017. Because only those microorganisms sent to EFSA in the frame of notifications for market authorisation are considered for the QPS status, it is important to stress that the QPS list is not exhaustive and that, therefore, it cannot be considered as a positive list.

The QPS Panel Statements contain the evaluations of the new notifications of microorganisms for a possible QPS status. It also contains a screening of the literature published during the previous 6 months period concerning possible new safety concerns related to the TUs included in the QPS list. The data identified in the literature are used to decide whether any TU may remain or not in the QPS list and whether the qualifications need to be revised. Since 2016, the literature update is performed by extensive literature searches (ELS).

The QPS Opinion contains an updated QPS list and summarises the results of the 3-years ELS on the QPS TUs, together with an update of the QPS granting process. Currently, the 2016 update of the recommended QPS list (including additions from Panel Statements) includes 95 TUs distributed as follows: 60 species of Gram-positive non-sporulating bacteria (mainly lactic acid bacteria), 13 *Bacillus* species, two Gram-negative bacteria, (*Gluconobacter oxydans* and *Xanthomonas campestris*, both only for food additive production), 15 yeast species and three virus families used as plant protection products (plant viruses *Alphaflexiviridae*, *Potyviridae*, insect viruses *Baculoviridae*; EFSA BIOHAZ Panel *et al.*^2017^).

## CONCLUSION

The QPS approach is a valuable tool for microbial safety assessment, which is in use within EFSA. The QPS approach allows speeding up the safety assessment process of microorganisms and their related products, providing a simplified evaluation. The ‘QPS status recommended biological agents in support of EFSA risk assessments list,’ initiated in 2007, is maintained through a process of continuous monitoring of possible safety concerns and expanded by the inclusion of new microorganisms. The maintenance is done by running an extensive literature search every 6 months to update the information behind the QPS TUs. The addition of new TUs to the QPS list depends on the evaluation of new notifications to EFSA through application dossiers. Both exercises are included in Panel Statements that are published every 6 months. Each of these Panel Statements and the Scientific Opinions, published every 3 years, are opportunities to update the QPS concept/approach. Recent examples are: (i) the application of the QPS concept to safety assessment of GMMs used for production purposes (EFSA BIOHAZ Panel *et al.*2018a), and (ii) the clarification of the qualification ‘for production purposes’ when microbial biomass is used for food and feed products (EFSA BIOHAZ Panel *et al.*2018b). In addition to its usefulness for risk assessment evaluation within EFSA, the QPS concept is becoming entrenched in the scientific literature, as evidenced by the increasing number of citations referring to QPS over time.

## Footnotes

3 Published as updates to the Scientific Opinion (EFSA BIOHAZ Panel, 2016) available at https://doi.org//10.2903/j.efsa.2017.4664 and, as of January 2018, also as supporting information linked to every Panel Statement available on the Knowledge Junction at https://doi.org/10.5281/zenodo.1146566.

## Notes

This paper is dedicated to Günter Klein (1964–2016), former member of the EFSA Working Group on QPS,^1^ who passed away on 22 December 2016.
